# Facial Aesthetics in Young Adults after Cleft Lip and Palate Treatment over Five Decades

**DOI:** 10.1038/s41598-017-16249-w

**Published:** 2017-11-20

**Authors:** K. Sinko, J. Cede, R. Jagsch, A. L. Strohmayr, A. McKay, W. Mosgoeller, C. Klug

**Affiliations:** 1 Clinic for Cranio-Maxillofacial and Oral Surgery, Medical University Vienna, Vienna, Austria; 2 Department of Applied Psychology, Health, Development, Enhancement, Intervention, Faculty of Psychology, University of Vienna, Vienna, Austria; 3 Bernhard Gottlieb University Clinic of Dentistry, Medical University, Vienna, Austria; 4 Institute of Cancer Research, Medical University Vienna, Vienna, Austria

## Abstract

Cleft Lip and Palate (CLP) - a common facial malformation in newborns – is typically corrected by surgical intervention to allow for normal speech development, psychosocial adjustment, and facial attractiveness. The long term treatment outcome can be evaluated after a number of years, possibly in adulthood. We investigated the aesthetics of the nasolabial region by subjective ratings. To compare various surgical approaches we recruited 12 raters to evaluate 429 patients. Expert and lay raters judged photographs from patients, who have completed treatment with one of three different surgical strategies performed in our institution over 50 years. Facial photographs were cropped, presented to the raters in a randomized sequence, and judged by the raters on a 5 point Likert scale. The subjective ratings between the raters revealed a fair to substantial inter-rater reliability. The average ratings of the surgical outcome improved continuously over the investigated 5 decades. Despite possible differences between raters and rater groups this overall result was consistently seen in the gender groups (male/female), or expertise related groups (expert/lay). Our analysis revealed that patients with bilateral CLP scored worse than patients with unilateral CLP when treated in the fifties; more recently treated patients of both groups scored similarly.

## Introduction

In the Caucasian population, about 1 in 700 newborns is born with a unilateral or bilateral cleft lip and palate (CLP). Whereas clefts of the lip are largely a cosmetic issue, clefts involving the palate pose a threat to the child’s development and physical well-being. During infancy, patients experience feeding difficulties; speech, hearing and dental problems occur as they grow older. Potentially life-long social and psychological consequences derive from the facial deformity itself^1,2^ and adversely influence the perception of facial attractiveness. An attractive face correlates with a number of positively perceived traits^3^. Aspects such as symmetry, averageness or a baby face affect the observer, regardless of his/her cultural background^4^, and can therefore disturb personal relationships and career^5^.

### Treatment of CLP

The core of any treatment plan is surgery. However, different centers use different surgical strategies and techniques which are subject to change over decades. A modern treatment method considers both function and aesthetics, and attempts to keep the number and impact of scars associated with the surgical intervention low^2^.

Two main surgical strategies (schools) are relevant. The first - more common in Anglo-American countries - proffers a so called “all in one” approach. Within the first year of life all anatomical structures that developed incompletely (lips, hard palate, soft palate) are repaired. An early closure of the soft and hard palate (until the age of 18 months) supports speech developing therapies and allows for better speech development. However, the surgery associated scars may impede on regular tissue growth and facial development.

Because a scar in the periost can cause dramatic growth abnormalities in the midface the second school puts more weight on bone growth. Therefore, only the soft tissue of the clefted area (lip, soft palate) is closed in the first year of life, while the structures covered by periost are not touched. The hard palate is closed later between the age of four and twelve^6^. This strategy ameliorates denudation of the periost in the first years of life, allowing for more time with less impact of surgery associated scars^7^.

In the last 20 years mixed models became widely adopted. They merged the different methods and treat the patient at the pre-school age between 3 month to 3 years.

In Vienna, starting from the Veau method^8^ Hollmann systematically explored alternative and improved strategies^9,10^. Based on animal research, he developed the “Vienna protocol”^11^ in the 60′s. This technique was characterized by a very early closure of the vermillion (Lip-adhesion) within the first weeks of life and the closure of the soft palate before the baby starts to speak between 6 and 12 months.

The remaining parts of the lip and the nose were treated, when the patient was about 4 years old; the hard palate was closed at the age of 6 before the patient started with school. Typically, the floor of the nose was built with the secondary osteoplasty to close the alveolar ridge between 8 and 12 years of age.

This approach allowed for a fair speech development, without any dramatic growth disturbance of the midface^12^.

The overall principle of Hollmann’s strategy was not to dash periost tissue too early, thereby avoiding growth inhibition as much as possible. However, this strategy requires an average of 5 surgical interventions over 12 years.

In the 90′s, the Hollmann’s scholars modified this strategy. Lip adhesion in the first weeks of life and upper lip-nose correction at the age of 4 were abandoned in favor of whole lip and floor of nose closure between the ages of 3 and 6 months.

### Treatment evaluation

While there are internationally validated outcome measures for speech and facial growth in CLP patients, there is no such system for assessing outcomes in post-surgery facial aesthetics^13^. The treatment of CLP is very complex, the outcome evaluation is equally complex in turn. It is very difficult to define a valid evaluation strategy and outcome relationship between the various surgical strategies. Many variables influence the outcome, e.g. the severity and extent of the malformation, the surgical strategy and the individual surgeon, the timing with respect to the patients age, etc.

It is an open question whether the judgements of experts or lay persons are more relevant. There may be large differences between the judgement of experts in the field or lay raters^14^. In contrast, Meng *et al*.^15^ describe similar ratings between experts and lay raters. The Eurocleft project systematically applied an evaluation strategy based on the Likert scale to provide an extensive overview over the various treatment strategies available in Europe. Shaw *et al*.^16^ describe, that in 201 centers, 194 different treatment concepts are practiced. A similar project in the USA^17^ reported 169 cases with unilateral CLP from five centers, and described significant differences between centers. When evaluating midface growth with the GOSLON score^18^, the centers performing primary osteoplasty scored worst. In contrast, no such differences were described in respect to the nasolabial appearance^17^. We found no comparable studies to investigate the change of outcomes over several decades. While most studies compared outcomes from different centers, we aimed to compare the aesthetic outcome of different decades concerning one center. The treatment outcome is highly variable due to a number of confounders. To obtain robust evaluation results large cohorts are needed^19^. We mobilized our institution archives and compared three surgical strategies in over 400 cases treated over 5 decades, and tested differences in outcome related to gender and expert status.

## Material and Methods

Our clinic is, and has been, a team care center for CLP-management for the east Austrian region for many years. We evaluated the photographic records from 429 patients (283 unilateral and 146 bilateral) with CLP, who were treated in our center from the 50′s to recently. The study was approved by the ethics commission of the Medical University Vienna, Austria (No: 1963/2016). All methods were carried out in accordance with the ethics commission’s approved vote. Specifically, we obtained informed consent for study participation, and we obtained informed consent to publish the images in an online open-access publication. In addition the pictures of patients’ faces presented to the raters and selected for publication were cropped and masked to render them unidentifyable. The cropping avoids full face presentation, the method complies to the US guidance regarding methods for de-identification of protected health information in accordance with the health insurance portability and accountability act (HIPAA) privacy rule.

Over the observed five decades, three generations of surgeons performed three different surgical strategies. Every strategy was typically applied by two or three senior surgeons with specific training. Any new strategy was gradually introduced and established.

### Patient & Photographs

We retrieved photographs from patients aged between 15 and 30 years, born between 1951 and 2001. They were treated in our center in the age between 2 weeks and adulthood. The photographs documented the original malformation and the post treatment photographs. For evaluation we used the most recent photographs documenting the long term outcome of the treatment. Patients with isolated cleft lips or patients treated primarily in other centers were not recruited.

We have no reliable information how many photographic documentations got lost over the decades and therefore were not included into the data pool.

After digitalization, the photographs were cropped as described previously^20^ and adjusted for contrast and lightness. Because the photographic images have been archived in total darkness, we did not have to consider possible fading in elderly analogue photographs.

**Figure 1:**
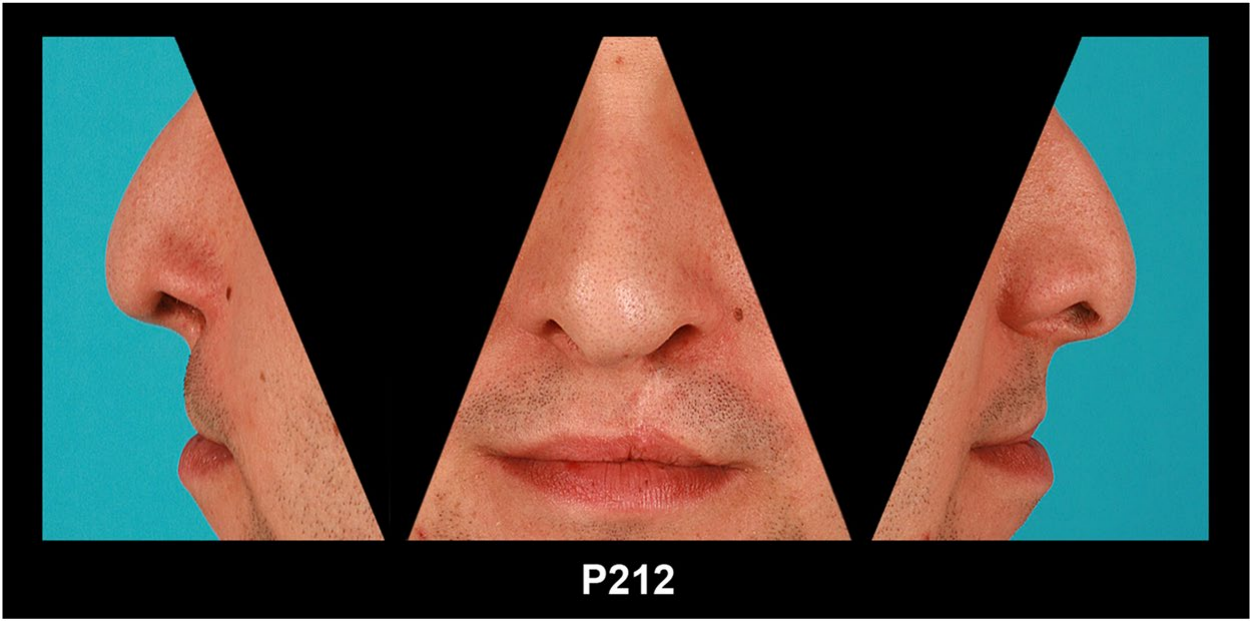
Example set with two lateral and one frontal view of the nasolabial region, of patient P212, as presented to the raters.

Of each patient (age between 15 to 30) a set of three cropped images was produced and labelled with a number only. Each set contained three views, i.e. lateral left, frontal, lateral right (Fig. 1) of the nasolabial region in adulthood (15–30 ys), i.e. years after the primary treatment.

### Raters

It is a matter of debate whether or not the face-perception differs between experts in the field or lay persons, between females and males^14,20^. Because an average of the scores of three raters appears to be sufficient for a substantial reliability in rating nasolabial appearance^21^, we recruited female and male raters, who were experts in the field or lay persons to form a panel of 12 raters altogether.

The six expert raters were involved in CLP treatment for many years (maxillofacial surgeons, orthodontist, speech language pathologist, and psychologist). The six lay persons were students (law, economy, sociology, dentistry) not familiar with CLP treatment.

### Procedure

To be comparable with the Eurocleft and Americleft studies for the documentation of the rater score, we used the method of Asher-McDade *et al*.^22^, which can be used reliably with three or more observers.

After ten openly discussed sets (warming up phase), the cases were presented to the raters at random using a power-point-presentation. After the warm-up, the raters judged four variables (nose, profile, symmetry of the nose, vermillion border) independently, i.e. without interaction between the raters. A typical single assessment of one photographic set took a few seconds. Ratings were documented for each case on a five point Likert scale.

The documented ratings were entered into a computer spread sheet twice. The second entry was subtracted from the first to receive zero as result in the case that both entries are identical. Errors were corrected, the completed data-set was statistically analyzed. The closed data set is available from the corresponding author.

### Statistics

We used the software package IBM SPSS for Windows Version 23. Statistical significance was set at p ≤ 0.01. Describing sociodemographic variables of patients was done using absolute and relative frequencies. Univariate comparisons (e.g. males with vs. without beard; Caucasian vs. non-Caucasian) were conducted using *t*-tests for independent samples. For simultaneously analyzing main group and main time effects as well as interaction effects, the General Linear Model (GLM) for repeated measures was used, given that prerequisites proved fulfilled (a normal distribution of subsamples, homogeneity of variances and co-variances). In case of significance, additionally effect sizes partial eta^2^ scores were provided (low effect size: eta^2^ > = 0.01, medium: eta^2^ ≥ 0.06, high: eta^2^ ≥ 0.14).

Inter-rater reliability for dyads of raters was calculated by means of Cohen’s kappa with linear weighting using the Landis and Koch^23^ interpretation of kappa scores: <0.20 slight, 0.21 to 0.40 fair, 0.41 to 0.60 moderate, 0.61 to 0.80 substantial, 0.81 to 1.00 almost perfect agreement. Aggregated means of Cohen´s kappa were then presented for the features nose, symmetry, vermillion border and profile, respectively.

## Results

### Patients

Table 1 and Table 2 describe the demographic details of the 283 unilateral and 146 bilateral CLP patients enrolled.

**Table 1:** CLP patients.

	Unilateral CLP (N = 283)		Bilateral CLP (N = 146)	
Cleft side	180 Left (63.6%)	103 Right (36.4%)	—	—
Race	260 Caucasian (91.9%)	23 other (8.1%)	140 Caucasian (95.9%)	6 other (4.1%)
Males with beard	125 without (76.7%)	38 with beard (23.3%)	67 without (72%)	26 with beard (28%)

**Table 2:** Number of analyzed cases per decade.

Patients	Unilateral CLP			Bilateral CLP		
treated	Male	Female	Total	Male	Female	Total
50 s	17	11	28	18	11	29
60 s	44	35	79	18	18	36
70 s	37	23	60	26	6	32
80 s	29	22	51	14	7	21
90 s	36	29	65	17	11	28
Total	163	120	283	93	53	146

In patients with UCLP, during the 50′s the mean age was 21.6 yrs, in the 90′s the mean age was 18.12 yrs. The patients age means from the other groups were in between these two means. The overall mean of all patients in all decades was 19.66 ( ± 3,531 STD). The effect sizes between the decades was medium to small (eta² = 0,080)

In patients with BCLP, the mean age in the 50′s was 21.76, and 19.79 in the 90′s. The overall mean was 20.94 ( ± 3.828 STD), The effect size was even smaller (eta² = 0,050).

### Inter-rater reliability

**Figure 2:**
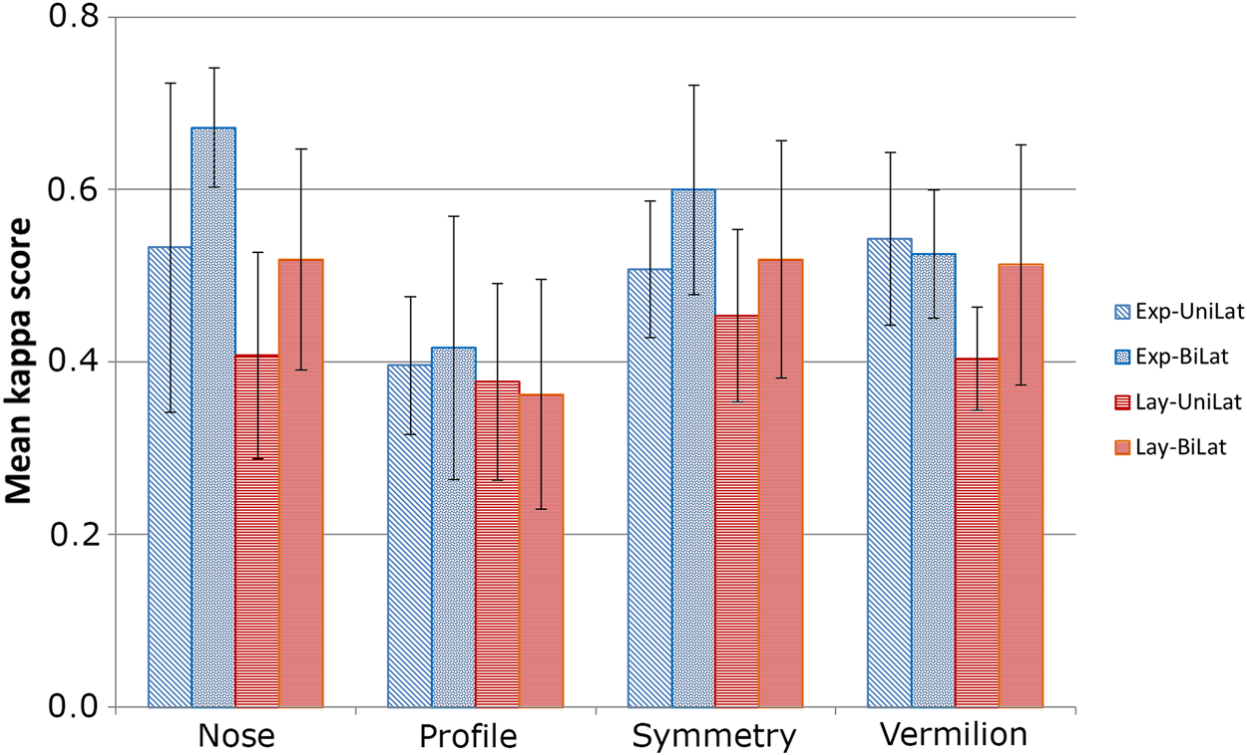
Means of Interrater reliability ( ± SEM) between the rater subgroups and the rated items (nose, profile, symmetry, vermillion border). Except for “profile” the interrater reliability was generally moderate (0.4–0.6), and substantial in two cases (≥0.6).

Figure 2 reveals the average of 15 possible dyads between the 6 experts and 6 lay raters for unilateral and bilateral CLP patients, for each of the four rating items (nose, profile, symmetry of the nose, vermillion border). The range of the average ratings was from 0.362 (fair) to 0.672 (substantial) for the respective variable and rater groups (expert and lay).

There was a trend for the inter-rater reliability being higher in expert raters compared to lay raters. Except the rating of the profile there was a trend for higher inter-rater reliabilities for the judgement of the nasolabial appearance after the surgical correction of CLP.

### Ratings

The comparison of race (Caucasian faces and others) revealed a small difference. Caucasian received better scores than non-Caucasians, however the effect size was very small (p = 0.020, effect size: eta² = 0.019). In view of the small group size with respect to beard and race we did not go into more detailed analysis.

**Figure 3:**
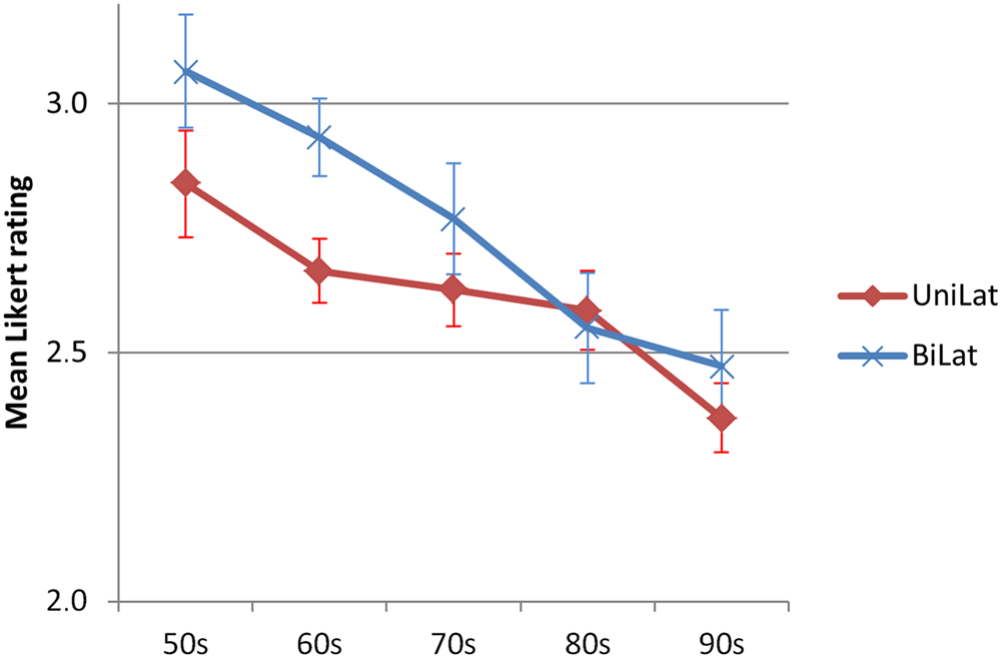
The aesthetic outcome (mean of 12 raters ± SEM) of uni- and bilateral CLP treatment improved continuously over the five decades.

Figure 3 shows a continuous improvement of the pooled data over the decades. This improvement is observable for both unilateral and bilateral CLP patients.

### Difference between raters

**Figure 4:**
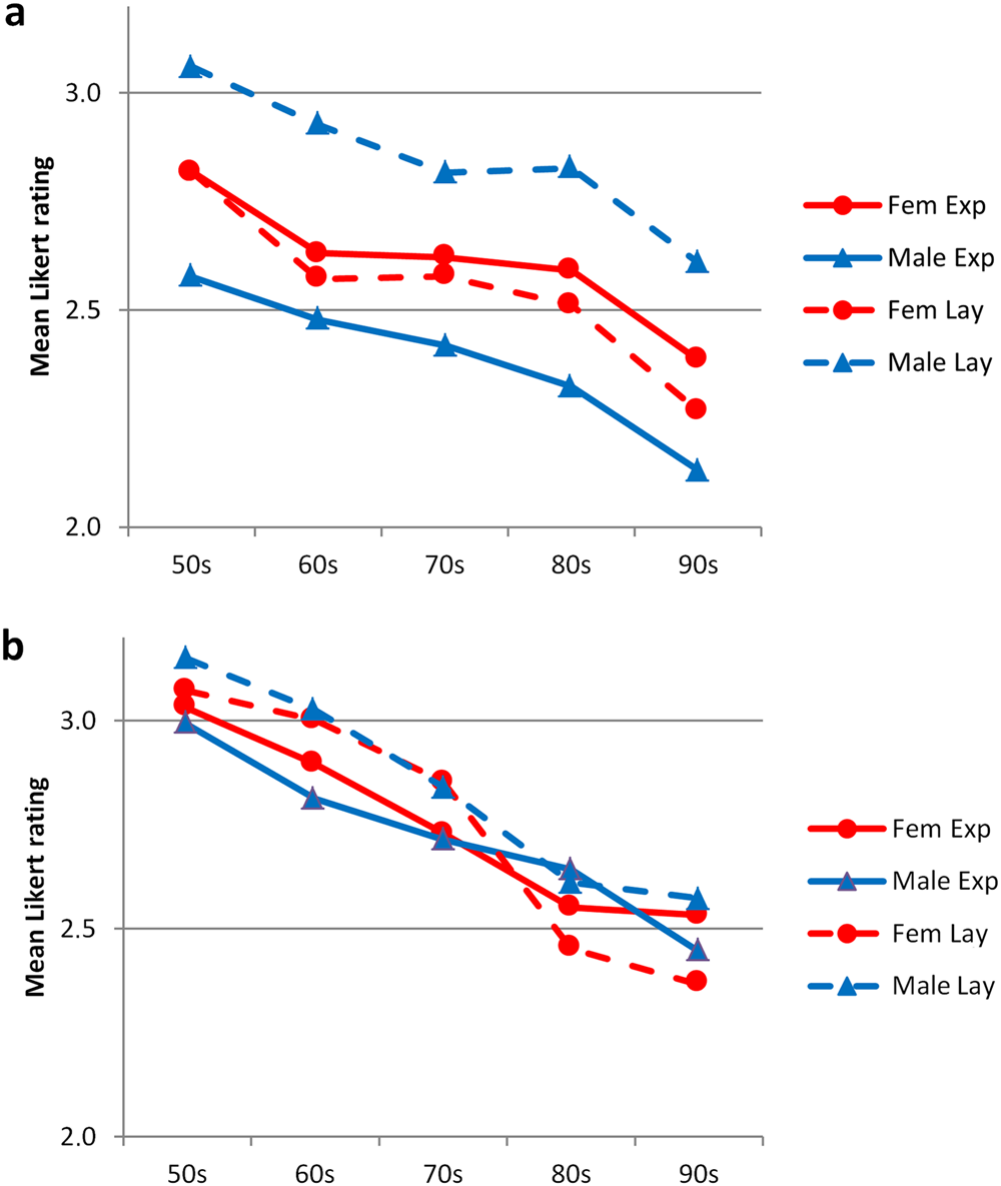
The aesthetic outcome of uni- and bilateral CLP treatment from 1951 to 2001 improved continuously irrespective different experience levels and the gender of the rater. [(**a**) - unilateral CLP; (**b**) - bilateral CLP] To increase the readability error bars were omitted.

Figure 4 reveals the means of expert and lay, male and female raters. Interestingly the judgments of each subgroup revealed similar improvements of the outcome over the decades. We observed different levels of the ratings depending on the raters gender and/or experience. These different levels were obvious when judging faces after the repair of unilateral CLP (Fig. 4a), they were hardly recognizable in patients with a corrected bilateral CLP (Fig. 4b).

In the evaluation of unilateral CLP patients the female raters scored very similarly, irrespective of the raters’ experience. The male lay raters were most critical, the male expert raters were the least critical (Fig. 4).

### Differences between patients

**Figure 5:**
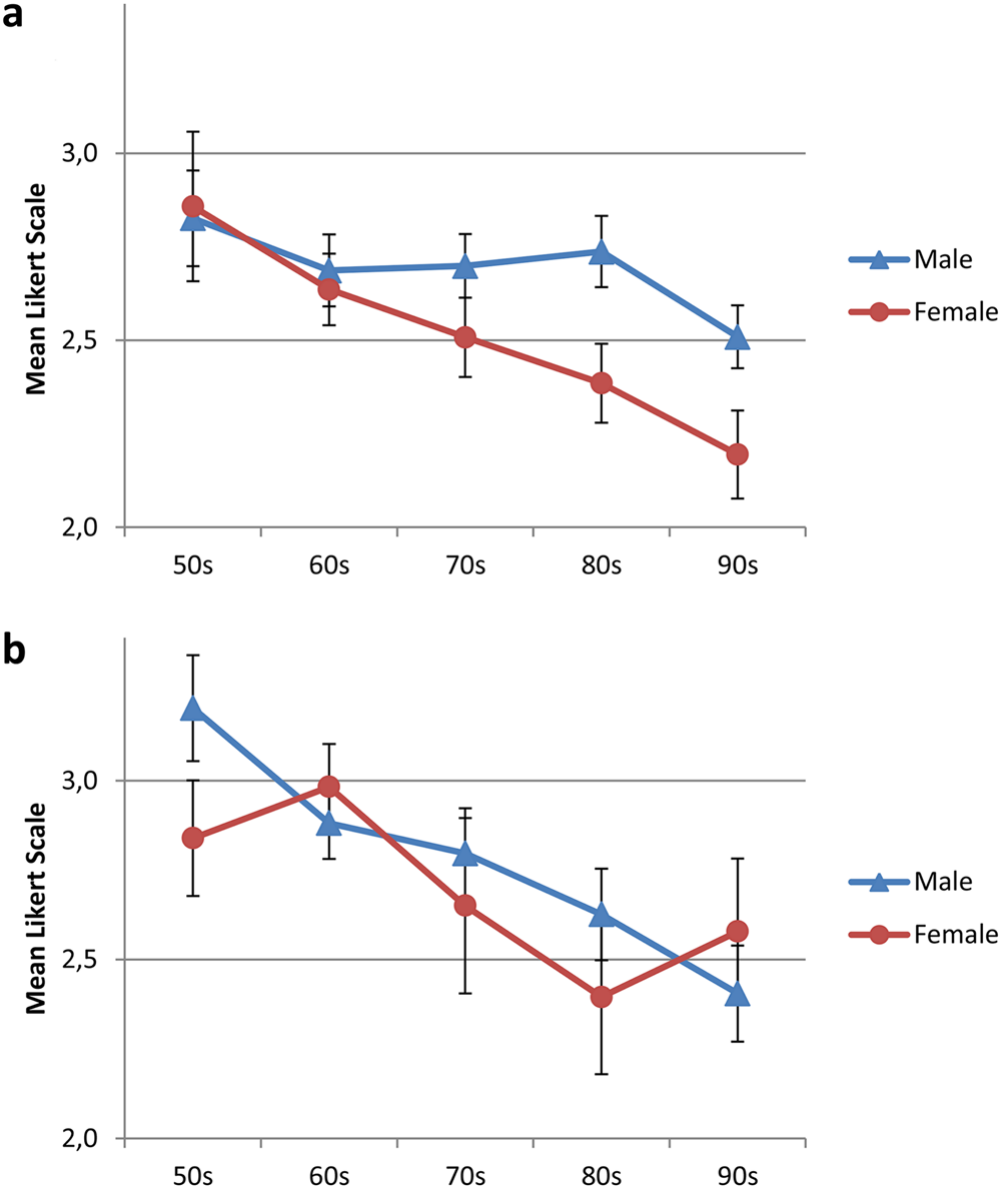
Male and female patients with a uni- and bilateral CLP, improved over five decades. [(**a**) - unilateral CLP; (**b**) - bilateral CLP]. Means ( ± SEM) of 12 raters.

Figure 5 compares the ratings between male and female patients over the five decades, in patients with repaired unilateral and bilateral CLP respectively.

The improvements over the decades were observable in both genders. In both patient groups - unilateral and bilateral - female faces tend to get better scores than male.

### Surgical methodologies

**Figure 6:**
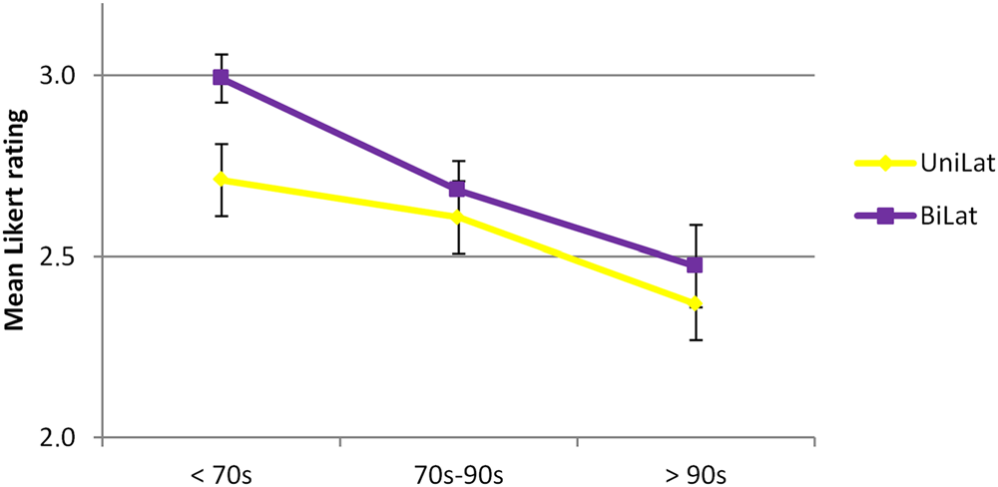
Outcomes of the three different treatment regimes; before 1970, from 1970 to 1990, and since 1990. Means ( ± SEM) of 12 raters.

Figure 6 summarizes the 12 raters judgements of three different therapeutic strategies. The Veau method was practiced prior to 1970. The Hollmann method was practiced from 1970–1990, followed by the modified Hollmann method, which has been employed since 1990.

As expected both patients with unilateral and bilateral CLP improved over the decades. Faces with repaired unilateral CLP scored generally better than those with bilateral CLP. The improvements in both unilateral and bilateral CLP patients were highly significant (p = 0.007; p = 0.001; respectively). However the effect size was small, e.g. (eta² = 0.026) in bilateral CLP.

## Discussion

Our data demonstrates a continuous improvement of the postoperative nasolabial appearance over the decades (1950ies to 2000), which may be attributed to various aspects.

### The rating procedure

Our methodology to evaluate the aesthetic long term outcome of therapeutic interventions, employed subjective ratings by professional and lay persons as described before. To consider different possible perceptions by male and female raters, we balanced the ratio between male and female raters. The assessment of a single case with four ratings took each rater less than one minute. According to Mosmuller *et al*.^24^, this is a reliable and cheap method to rate treatment outcomes non-invasively.

Our rating methodology - clinical photograph evaluation - was among the main approaches described by Sharma *et al*.^13^. We used the five point Likert scale as it has been evolved from the Asher-McDade system^22^. Although the rating scale is a matter of scientific debate^25^. a 5 point Likert scale may be superior to a 3 point scale because of better resolution^13^.

Although semi-analog scales are discussed^25^ the big studies like Americleft^26^ and Eurocleft^27^ used a 5 point Likert scale. In view of the parameters’ natural variability we found a finer scale less useful, and adopted a 5 point scale, also to be comparable with the majority of the previous reports.

While Kocher *et al*.^28^ claimed that the rating of full faces is not biased by the overall facial attractiveness, Sharma *et al*.^13^ found that cropped photographs were more representative than full faces. However, to standardize the presentation of available post-surgery photographs from different decades, we cropped images to help the raters focus on the nasolabial region.

The presentation of the cropped image sets was randomized to avoid a possible anchoring on particular sets and a bias due to a specific presentation sequence. Furthermore, because the rating of frontal views alone may not be sufficient^29^ we analyzed a standardized mix from lateral and frontal views i.e. lateral right, frontal, lateral left (Fig. 1).

The cropping allowed for a masking of the age of the photographs, whilst also masking hints related to fashion and style, specific for any of the investigated decades.

Because our presentation material was produced from the clinical documentation stored as color slides kept under dark condition, no fading of old slides - which might influence the raters – were recognizable.

The cropping of the photographs, the adjustment in brightness and contrast, and the limited time to focus the raters attention on the four categories, also aimed to decrease a confounder, i.e. a possibly different photograph quality related to different equipment used for their production over the decades.

### Rater variability

It is a matter of debate whether or not expert or lay raters reach different conclusions^14^. To account for possible differences attributable to the raters’ experience or gender, we analyzed the respective subgroups. Our data indicated a recognizable difference between male experts and male lays, which was not found between female experts and female lay raters (Fig. 4). When analyzing the inter-rater reliability with regard of different views such as the nose, profile, symmetry, and vermillion boarder, our data did not substantially differ between lay and expert raters (Fig. 2). The observed subtle differences indicate, that the overall outcome may depend on the assembly of the raters panel.

In contrast, the observed variabilities between ratings of facial regions (profile, nose) indicated that the differences between facial regions may contribute to reported differences between expert and lay, female and male raters (Fig. 2).

Our finding in the subgroup analysis on a considerable difference between male experts and male lay (Fig. 4) may have occurred as a result of chance. It may explain some of the controversies related to the issue whether or not there is a difference between expert and lay raters.

### Discussion of Overall results

The treatment outcome depends on a complex interplay of the severity of the malformation, surgical technique and surgeons experience, individual patients factors, ad-on treatments, etc. Irrespective of possible rater related bias, our overall analysis as well as the rater subgroup analysis reveals a continuous improvement of the outcome over decades. (Fig. 3).

Figure 4 shows this very same trend consistently for each of the rater group. Every group rated post-surgery faces from the 50′s worse than the most recent cases.

Interestingly, in the 50′s and the 60′s, the results for bilateral CLPs were rated recognizably worse compared to the surgery outcome of unilateral CLP faces; this was not observed in more recent cases. It is possible that the surgical techniques improved to the extent that our raters documented no considerable difference since the 80′s. The outcome for both patient groups, either with unilateral or bilateral CLP faces advanced over the decades significantly (p = 0.007; p = 0.001; respectively; Fig. 3).

Another subgroup analysis that revealed differences, comparing ratings given to male patients relative to those given to female patients (Fig. 5). The improvement over the decades was not always parallel for male and female patients. This may be due to the natural variability of the overall outcome.

The Asher-McDade Index has not been validated for the rating of older teenagers, adult patients, or patients with BCLP. This may limit the generalization of our observations. However, we randomized the sequence of case presentation during the rating procedure. Therefore we argue that observed differences between our groups or subgroups can reflect real differences in the outcomes over the decades.

## Conclusion

Irrespective of indications of high outcome variability, our study documents a continuous improvement of nasolabial appearance in patients after CLP treatment over the last 5 decades (1950 to 2000). Irrespective of possible differences between rater groups (male/female expert/lay) these findings are seen consistently between rater groups. In the last years uni- and bilateral CLP show similar outcomes.
